# Supplementary material for: Genome-Wide Analysis of Nuclear factor-YC Genes in the Tea Plant (Camellia sinensis) and Functional Identification of CsNF-YC6
Source: Int J Mol Sci. 2024 Jan 9;25(2):836. doi: 10.3390/ijms25020836 (PMC10815638; doi:10.3390/ijms25020836)
Supplement: Supplementary file 1 [file ijms-25-00836-s001.zip › ijms-2758513-supplementary.pdf]

SUPPORTING INFORMATION

Genome-Wide Analysis of *Nuclear factor-YC* Genes in the Tea Plant (*Camellia sinensis*) and Functional Identification of *CsNF-YC6*

Supporting Tables

Table S1. The primers used in the tests.

Primer name	Primer sequence（5' to 3'）	Used
CsNF-YC1	F: ATGGATCAGCACGGACAAC<br>R: TCAGAAAGCGGGCACAG	Cloning
CsNF-YC2	F: ATGTTCTGGACGTACCAACGC<br>R: TTAAAATCTCTGCCTCCTAAATGTG	Cloning
CsNF-YC3	F: ATGGATCAGCAAGGGC<br>R: TTAAGAATCTGCAGGTGGTT	Cloning
CsNF-YC4	F: ATGGATCAACAAGGGCA<br>R: CTAATTTAGCAGATCACATCTG	Cloning
CsNF-YC6	F: ATGGATCAGCAAGGGCAT<br>R: TTAAGAATCTGCAGGTGGTTGC	Cloning
CsNF-YC7	F: ATGAGGCAAGCAAGAAC<br>R: TCACATTTGGCCACC	Cloning
CsNF-YC8	F: ATGAGACAAGCAGGAACATATTCAG<br>R: TCAAGTCCCATAAGCCTTACCA	Cloning
CsNF-YC9	F: ATGGAGAACAACCAACAACA<br>R: TCACTTGGTAATGAGGTGC	Cloning
CsNF-YC6-OE	F: gagaacacgggggacgagctcATGGATCAGCAAGGGCATGG<br>R: gcccttgctcaccatgtcgacAGAATCTGCAGGTGGTTGCTG	Cloning
CsGAPDH-qPCR	F: GATAGTGTTCACGGTCAATGGA<br>R: GCAGCAGCCTTATCCTTATCAG	Internal reference
CsNF-YC1-qPCR	F: GCTTGCCTATCAACATATCCA<br>R: TGTTTTCCTCAGTATGATTCCA	Expressing
CsNF-YC2-qPCR	F: ATCTCCGCCGAAGCC<br>R: GCCCACAATCTCTACCCCT	Expressing
CsNF-YC3-qPCR	F: AACCATAGCCTTCCATTAGC<br>R: GATTCCAAGACCGTAGCG	Expressing
CsNF-YC4-qPCR	F: AGGAAAACAAAAGGAGGACA<br>R: AGGCGAATGCTGAGGTG	Expressing
CsNF-YC5-qPCR	F: AAGAGATGAGTTGAAAGAAGAGGG<br>R: TGGGGGGCTGAGGTTGT	Expressing

Primer name	Primer sequence（5' to 3'）	Used
CsNF-YC6-qPCR	F: CAGTTACGGGGGTGATGG<br>R: GCTGCTGGTGGATGTGCT	Expressing
CsNF-YC7-qPCR	F: TCTGCTGTTGTAGCCACTGAT<br>R: CTATGACTCCCACACCCCA	Expressing
CsNF-YC8-qPCR	F: GCCGCTGGCGAGGA<br>R: TCATCAGAGTTAGCAGAATCAGAA	Expressing
CsNF-YC9-qPCR	F: CATCACTCGCACTGACATTTTC<br>R: GCTCCCTCCGCTGGC	Expressing
AtActin2-qPCR	F: CTTGCACCAAGCAGCATGAA<br>R: CCGATCCAGACACTGTACTTCCTT	Internal reference
AtFLC-qPCR	F: CCTAATTTGATCCTCAGGTTTGGG<br>R: CCGACGAAGAAAAAGTAGATAGGCAC	Expressing
AtSOC1-qPCR	F: GCTCTCAGTGCTTTGTGATGC<br>R: AAGAACGTACTTGGAGCTGGC	Expressing
AtCO-qPCR	F: CCGGGTCTGCGAGTCATG<br>R: GGCATCATCTGCCTCACACA	Expressing
AtFD-qPCR	F: GCGCTAGGAAACAGGAATGCTTATACAAA<br>R: AGCTGTGGAAGACCGTTGAAGTGTG	Expressing
AtFT-qPCR	F: GAGACCCTCTTATAGTAAGCAGAGTTG<br>R: GGGAGTTCAAGTGAAAGAACCAAAGT	Expressing
AtLFY-qPCR	F: TTGGTGGTTTAGAGGGACTATTCG<br>R: GCCGTAAAACCTAACTCCGCTA	Expressing
AtAP1-qPCR	F: AACCAAGGCCACAATATGCC<br>R: CGGGTTCAAGAGTCAGTTCG	Expressing
AtDREB2A-qPCR	F: TGACCTAAATGGCGACGATGT<br>R: TCCAAGTAACTCAAGTCGTCG	Expressing
AtNCED3-qPCR	F: CAGCTTGTAGCTTTTGGGCTGTA<br>R: TAACAGAAACCAGCTGAGCTCGA	Expressing
AtRD29A-qPCR	F: GTTACTGATCCCACCAAAGAAGA<br>R: GGAGACTCATCAGTCACTTCCA	Expressing
AtABI1-qPCR	F: AGAGTGTGCCTTTGTATGGTTTTA<br>R: CATCCTCTCTCTACAATAGTTCGCT	Expressing
AtABI5-qPCR	F: CAATAAGAGAGGGATAGCGAACGAG<br>R: CGTCCATTGCTGTCTCCTCCA	Expressing
P1300	F: TCATTGCGATAAAGGAAAGGC<br>R: GCCACGATTTGACACATTTTTAC	Identification
CZ*CsNF-YC6*	F: ACGCACAATCCCACTATCCTTC<br>R: CGTTTACGTCGCCGTCCAG	Identification
hyg(280)	F: ACGGTGTCGTCCATCACAGTTTGCC<br>R: ttccGGAAGTGCTTGACATTGGGGA	Identification

Table S2. Tea plant *NF-YC* family members predict

Gene name	Sequence ID	Chromosome number	Chromosome location (bp)	HMM length	E value	AA	Molecular weight (KDa)	Isoelectric
CsNF-YC1	GWHPACFB011961	2	107147531-107148430	64	3.70E-21	260	29.32	5.85
CsNF-YC2	GWHPACFB013851	3	104792750-104795981	64	1.6E-20	177	19.96	5.38
CsNF-YC3	GWHPACFB026946	ChaUn10827 （TEA022201.1：chr4）	13103-13882	64	1.00E-21	228	25.41	5.77
CsNF-YC4	GWHPACFB020260	6	107576981-107578654	64	3.40E-21	247	27.59	6.05
CsNF-YC5	GWHPACFB024733	8	152348395-152350737	64	6.80E-21	309	34.58	5.45
CsNF-YC6	GWHPACFB033498	ChaUn9861 （TEA002102.1：chr10）	29812-42002	64	3.50E-21	480	52.95	6.01
CsNF-YC7	GWHPACFB005786	12	8344610-8345166	65	3.20E-21	153	16.43	10.01
CsNF-YC8	GWHPACFB009167	14	54319197-54320242	65	5E-21	126	13.67	9.3
CsNF-YC9	GWHPACFB031341	ChaUn5166 （TEA008802.1：chr15）	28410-29461	64	1.60E-20	230	25.51	5.33

Table S3. Basic information of all identified *CsNF-YCs* genes in tea plant.

Gene name	Identities	ORF（bp）	AA	pI	Molecular weight (KDa)	Instability Index	Aliphatic Index	GRAVY	SignalP	TMHs
CsNF-YC1	95.60％	747	248	5.85	27.98	71.06	68.87	-0.668	NO	0
CsNF-YC2	98.80％	501	166	5.04	18.54	51.09	83.43	-0.316	NO	0
CsNF-YC3	87.82％	780	259	5.88	28.74	71.24	64.48	-0.605	NO	0
CsNF-YC4	86.71％	1011	336	7.21	37.82	65.80	68.01	-0.619	NO	0
CsNF-YC6	99.86％	726	241	5.81	27.12	72.33	66.06	-0.648	NO	0
CsNF-YC7	94.34％	372	123	9.77	13.28	62.03	72.11	-0.341	NO	0
CsNF-YC8	98.60％	357	118	7.85	12.92	46.71	88.47	-0.226	NO	0
CsNF-YC9	97.69％	729	242	5.06	26.83	67.18	75.83	-0.378	NO	0

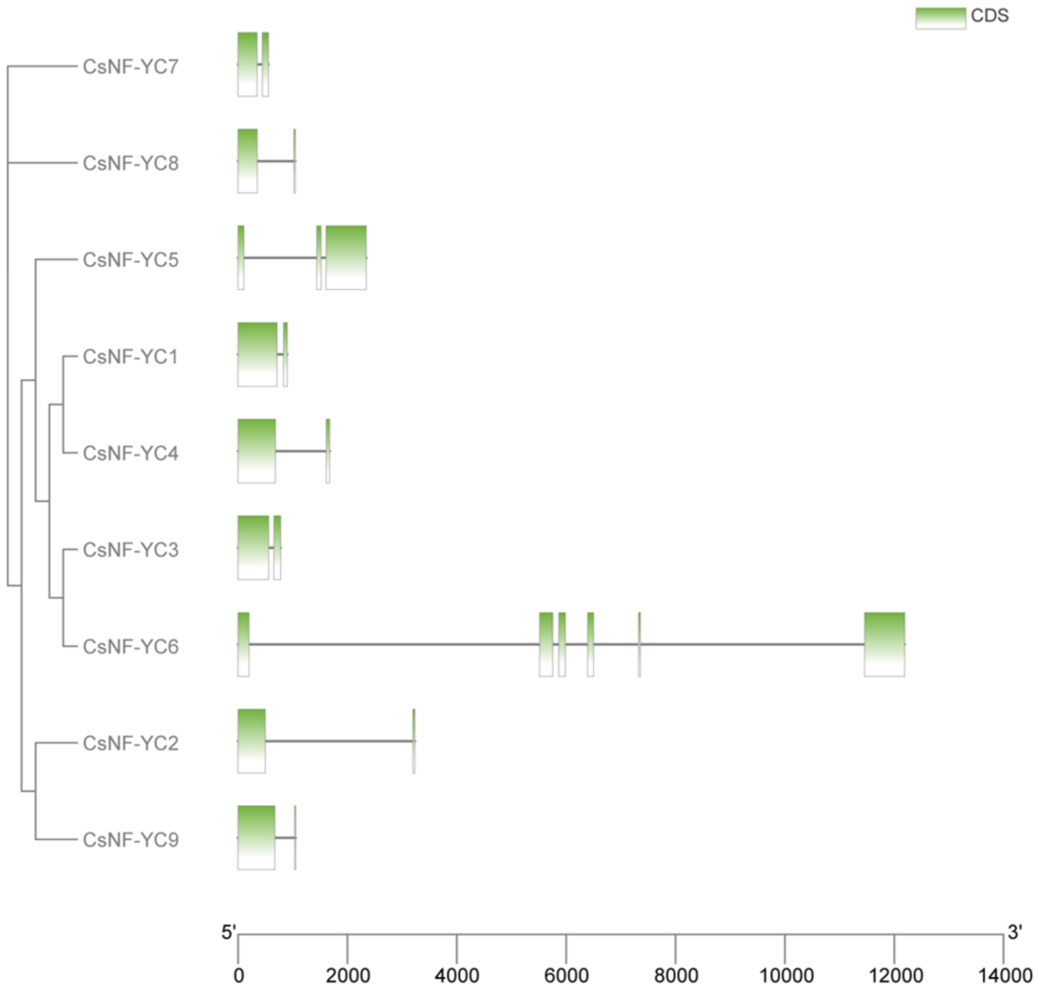

Figure S1. Exon-intron structure of *CsNF-YCs* genes.

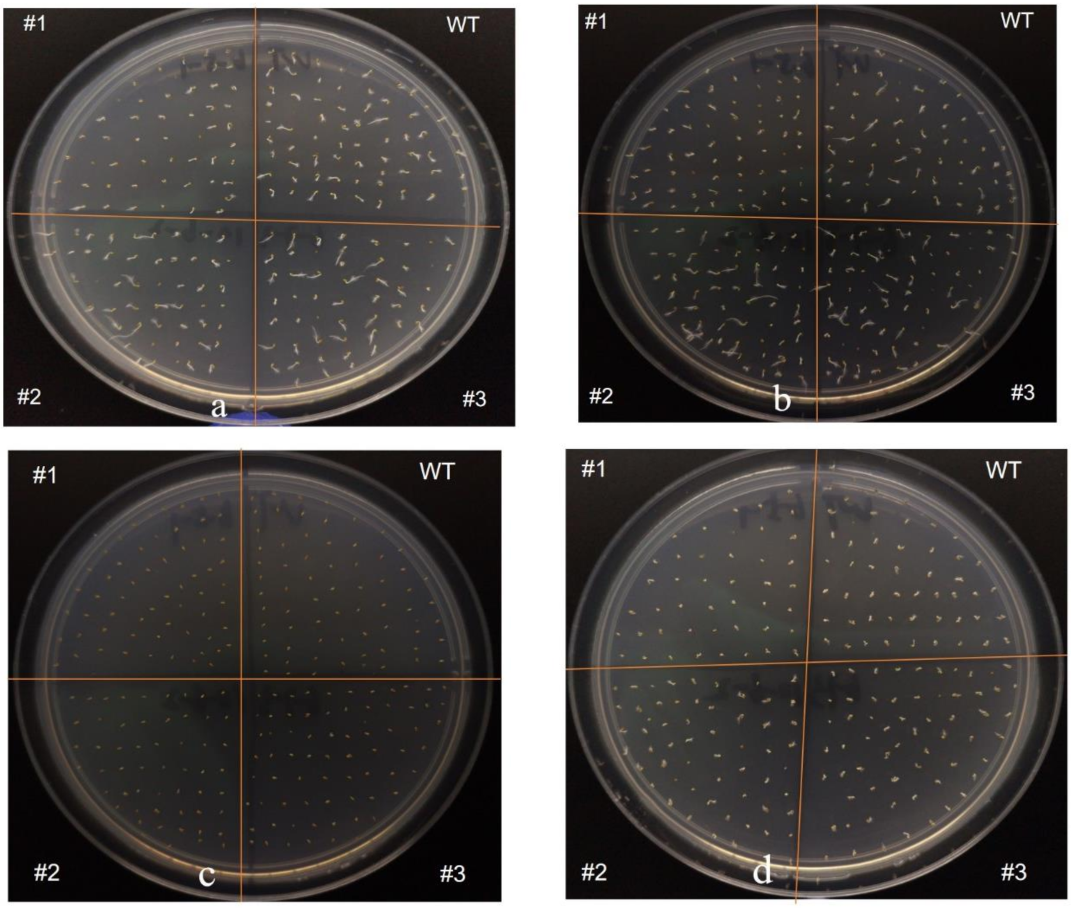

Figure S2. The phenotype of WT and three lines grown in 1/2 MS (a), 0.5 μM GA (b), 0.5 μM ABA (c) and 150 mM NaCl (d) medium for 4 days

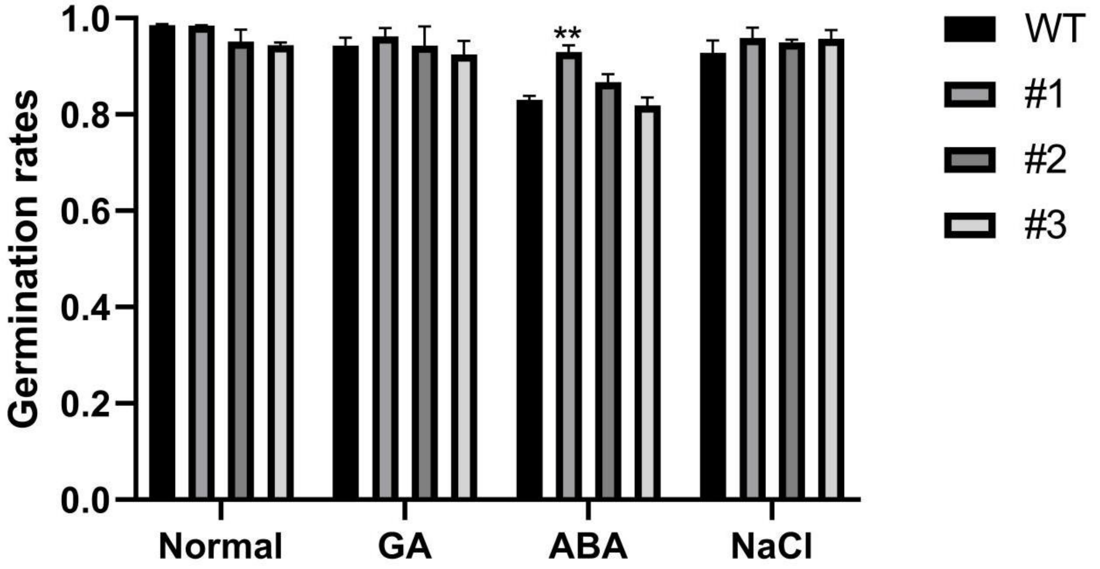

Figure S3. Germination rates of *CsNF-YC6-OE* Arabidopsis lines under different stress condition

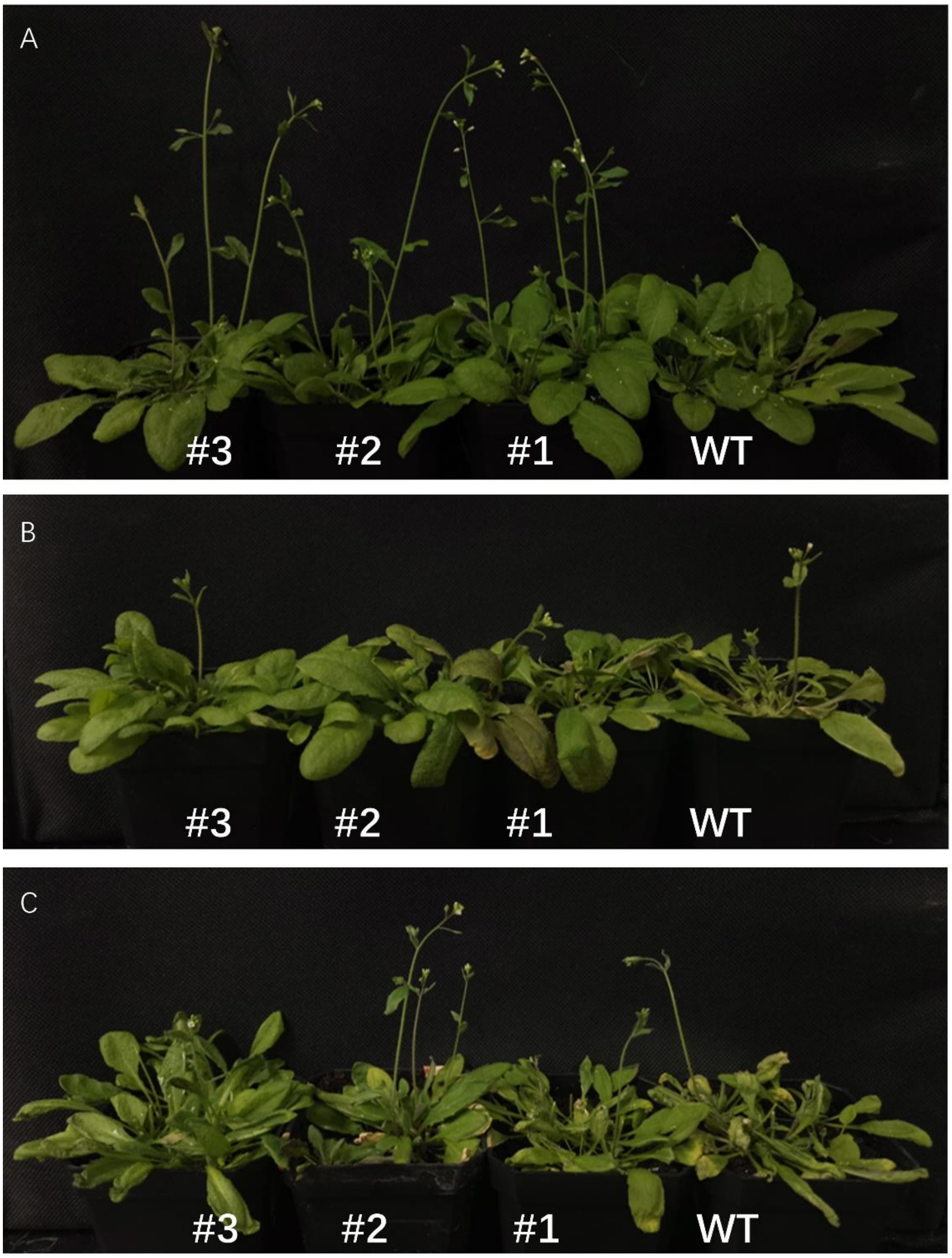

Figure S4. Phenotypic analysis of CsNF-YC6- overexpressed Arabidopsis thaliana lines and wild-type under different stresses: normal culture conditions (A), 200 mM NaCl treatment (B) and 15% PEG treatment (C).

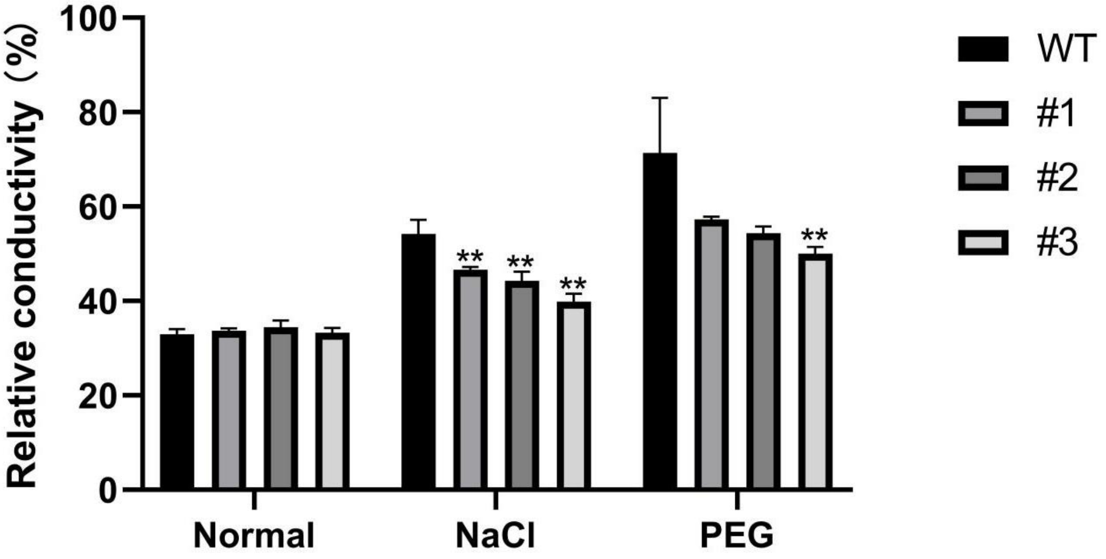

Figure S5. The relative conductivity of wild type and *CsNF-YC6-OE* Arabidopsis under stress.

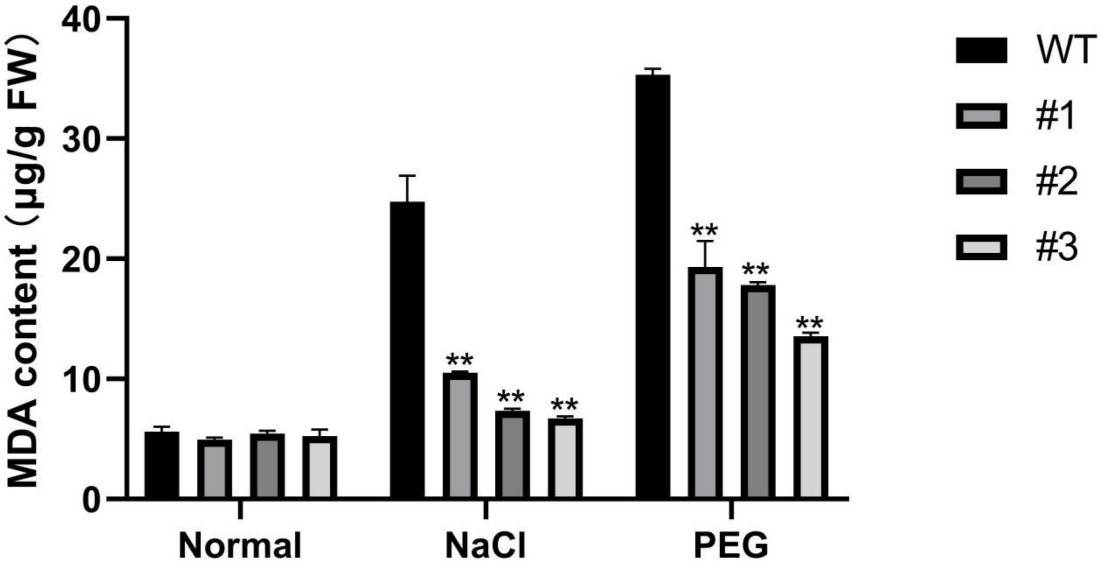

Figure S6. The MDA content of wild type and *CsNF-YC6-OE* Arabidopsis under stress.

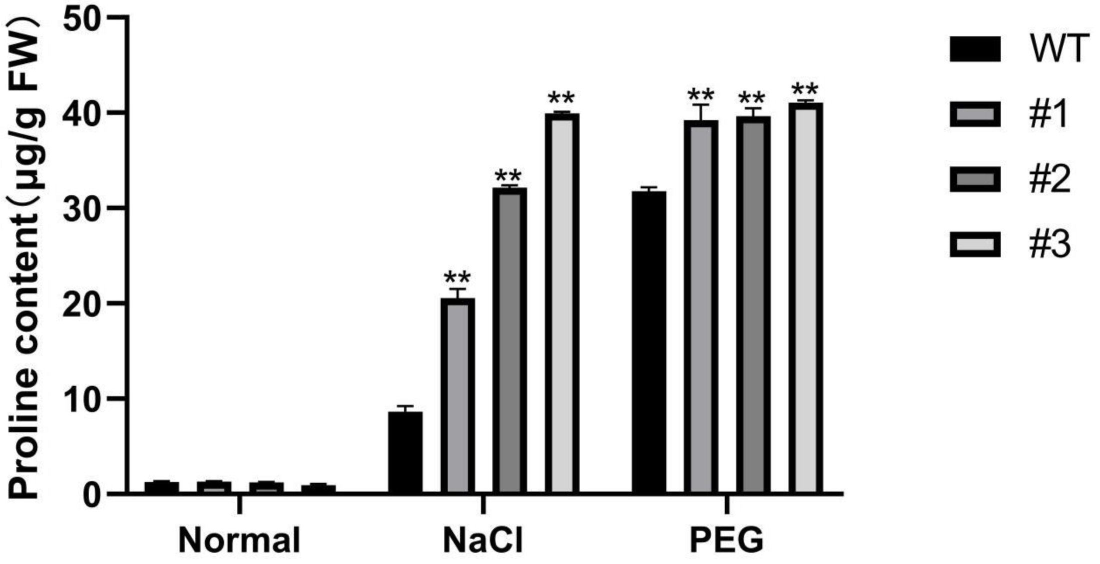

Figure S7. The proline content of wild type and *CsNF-YC6-OE* Arabidopsis under stress.

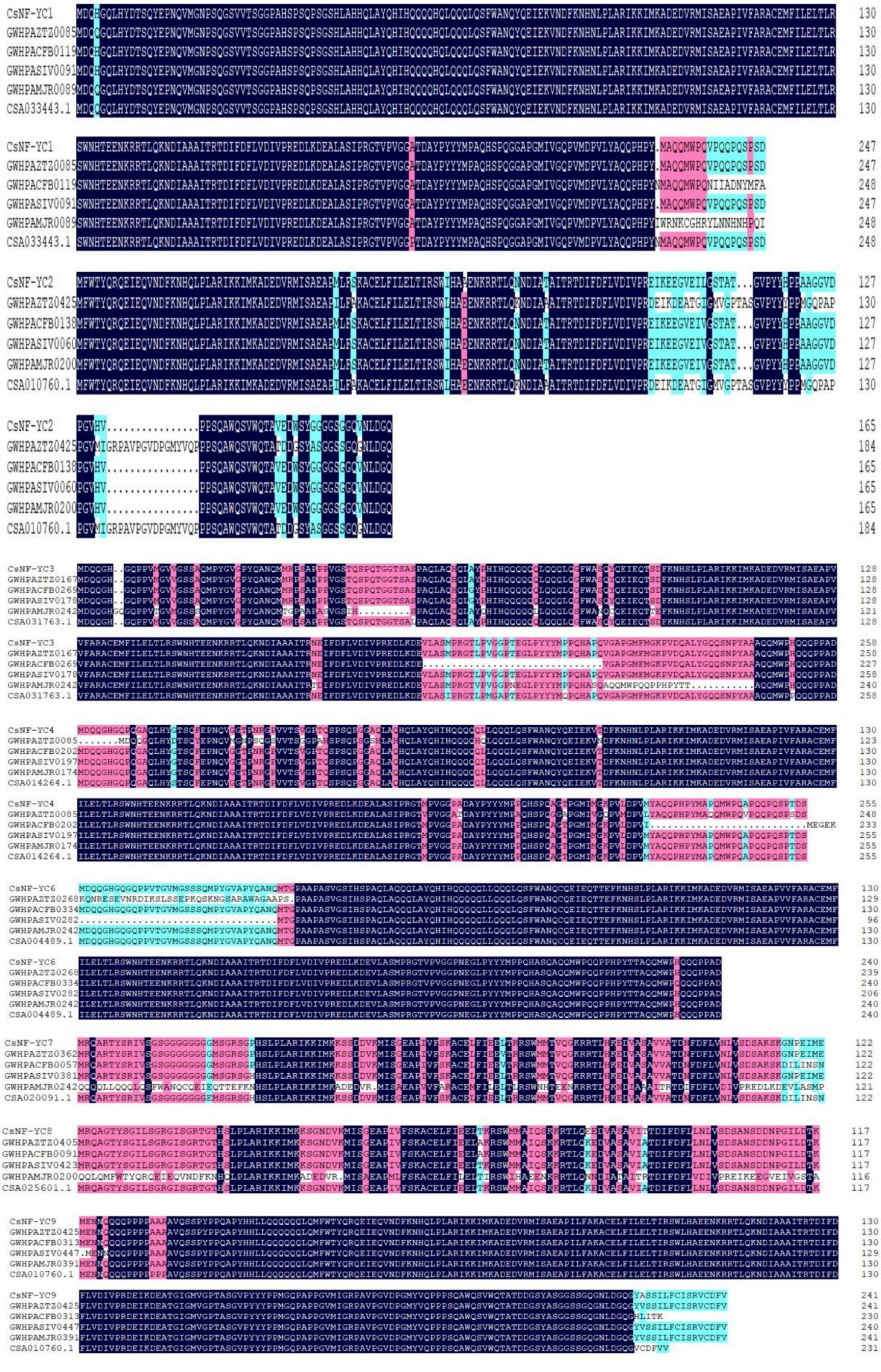

Figure S8. Polymorphism analysis of *CsNF-YCs*.
